# High-Throughput Mutagenesis Reveals a Role for Antimicrobial Resistance- and Virulence-Associated Mobile Genetic Elements in Staphylococcus aureus Host Adaptation

**DOI:** 10.1128/spectrum.04213-22

**Published:** 2023-02-23

**Authors:** Xiaoliang Ba, Marta Matuszewska, Lajos Kalmar, Jingyan Fan, Geng Zou, Desirée Corander, Claire L. Raisen, Shaowen Li, Lu Li, Lucy A. Weinert, Alexander W. Tucker, Andrew J. Grant, Rui Zhou, Mark A. Holmes

**Affiliations:** a Department of Veterinary Medicine, University of Cambridge, Cambridge, United Kingdom; b Department of Medicine, University of Cambridge, Cambridge, United Kingdom; c State Key Laboratory of Agricultural Microbiology, Huazhong Agricultural University College of Veterinary Medicine, Wuhan, China; d Department of Biochemistry, Faculty of Science and Engineering, Åbo Akademi University, Turku, Finland; e Cooperative Innovation Centre of Sustainable Pig Production, Wuhan, China; f International Research Centre for Animal Diseases (MOST), Wuhan, China; University at Albany

**Keywords:** antimicrobial resistance, host adaptation, MRSA, mobile genetic elements, transposon mutagenesis

## Abstract

Methicillin-resistant Staphylococcus aureus (MRSA) clonal-complex 398 (CC398) is the dominant livestock-associated (LA) MRSA lineage in European livestock and an increasing cause of difficult-to-treat human disease. LA-CC398 MRSA evolved from a diverse human-associated methicillin-sensitive population, and this transition from humans to livestock was associated with three mobile genetic elements (MGEs). In this study, we apply transposon-directed insertion site sequencing (TraDIS), a high-throughput transposon mutagenesis approach, to investigate genetic signatures that contribute to LA-CC398 causing disease in humans. We identified 26 genes associated with LA-CC398 survival in human blood and 47 genes in porcine blood. We carried out phylogenetic reconstruction on 1,180 CC398 isolates to investigate the genetic context of all identified genes. We found that all genes associated with survival in human blood were part of the CC398 core genome, while 2/47 genes essential for survival in porcine blood were located on MGEs. Gene *SAPIG0966* was located on the previously identified Tn*916* transposon carrying a tetracycline resistance gene, which has been shown to be stably inherited within LA-CC398. Gene *SAPIG1525* was carried on a phage element, which in part, matched phiSa2wa_st1, a previously identified bacteriophage carrying the Panton-Valentine leucocidin (PVL) virulence factor. Gene deletion mutants constructed in two LA-CC398 strains confirmed that the *SAPIG0966* carrying Tn*916* and *SAPIG1525* were important for CC398 survival in porcine blood. Our study shows that MGEs that carry antimicrobial resistance and virulence genes could have a secondary function in bacterial survival in blood and may be important for host adaptation.

**IMPORTANCE** CC398 is the dominant type of methicillin-resistant Staphylococcus aureus (MRSA) in European livestock and a growing cause of human infections. Previous studies have suggested MRSA CC398 evolved from human-associated methicillin-sensitive Staphylococcus aureus and is capable of rapidly readapting to human hosts while maintaining antibiotic resistance. Using high-throughput transposon mutagenesis, our study identified 26 and 47 genes important for MRSA CC398 survival in human and porcine blood, respectively. Two of the genes important for MRSA CC398 survival in porcine blood were located on mobile genetic elements (MGEs) carrying resistance or virulence genes. Our study shows that these MGEs carrying antimicrobial resistance and virulence genes could have a secondary function in bacterial survival in blood and may be important for blood infection and host adaptation.

## INTRODUCTION

Staphylococcus aureus is both a common commensal bacterium of the human nasopharynx and skin and an opportunistic pathogen, which causes diseases ranging from mild skin infections to life-threatening bacteremia, infective endocarditis, and toxic shock syndrome (1). Methicillin-resistant S. aureus (MRSA) strains are drug-resistant S. aureus that have acquired *mecA* or *mecC* genes, making them resistant to nearly all β-lactam antibiotics (2). Apart from being a leading cause of hospital and community infections, MRSA strains are frequently found to colonize and infect animals, especially livestock (3).

Clonal complex 398 (CC398), a livestock-associated MRSA (LA-MRSA) lineage, was first described in the Netherlands in 2005 (4). It has since increasingly spread worldwide, being found in more than 20 countries (5). In European countries, CC398 has become the dominant LA-MRSA in livestock as demonstrated in Danish pig farms, where the proportion of MRSA-positive herds has increased from <5% in 2008 to 90% in 2018 (6, 7). It is particularly concerning, as LA-MRSA CC398 has been associated with increasing numbers of human diseases, ranging from skin and soft tissue infections to invasive diseases, such as bloodstream infections, in both people with and without direct contact with livestock (8–11). Although it is believed that LA-MRSA generally causes less severe or complicated diseases than other MRSA lineages (12), possibly because human patients affected by CC398 are younger or stay for a shorter time in hospital (12), it is not thought to be less pathogenic as a human pathogen (13, 14). However, LA-MRSA CC398 rarely causes infections in livestock animals (15).

Previous studies have shown that the CC398 population structure can be described by two major groups: a methicillin-resistant livestock-associated clade that falls as either sister to (16) or within (17–19) a largely methicillin-sensitive clade prevalent in humans. These studies have shown that CC398 emerged in livestock around 1964 and that it was associated with the loss of the immune evasion cluster (IEC) and the acquisition of antibiotic resistance genes *tetM* and *mecA*. The investigation into the evolutionary history of the mobile genetic elements (MGEs) that carry these genes within CC398 showed that the Tn*916* transposon and the staphylococcal cassette chromosome *mec* (SCC*mec*) element are both common in livestock-associated CC398, whereas the φSa3 prophage is common in human-associated CC398. However, it is not fully understood how these population differences contribute to LA-CC398 causing disease in humans.

Due to the difference in CC398’s ability to cause disease in humans and livestock, further research is needed to understand the genetic basis of human adaptation, which can lead to diseases including bacteremia. The survival of bacteria in blood is determined by their ability to withstand the bactericidal activity of the innate immune system composed essentially of neutrophils, monocytes, and the complement system (20). Previous S. aureus blood survival experiments have shown an initial decrease and then a recovery in bacterial counts after up to 24 h of incubation (21, 22). In this study, we hypothesize that LA-MRSA CC398 interacts differently with human blood and porcine blood, which leads to a difference in its ability to establish and survive in the blood and cause bacteremia. In order to identify potential genetic signatures that are responsible for the difference, we employ transposon-directed insertion site sequencing (TraDIS), a high-throughput transposon mutagenesis method for functional phenotypic studies (23), to identify genes that are essential to LA-CC398 survival in human and porcine blood.

## RESULTS

### Construction and validation of transposon mutant libraries.

Highly complex *mariner* transposon mutant libraries were successfully generated in whole-genome-sequenced LA-MRSA sequence type 398 (ST398) strains S0385 and 09V (Table 1). These two closely related strains were selected from the LA-CC398 group isolated from humans to increase the power of the analysis and account for potential between-strain variation. Serially diluting the transposon mutant libraries and plating them on tryptone soy agar (TSA) supplemented with appropriate antibiotics determined a transposon mutant library size of about 1 × 10^9^ transposon mutants for both libraries. The process generated an S0385 transposon mutant library with a 100% cure (loss) of pMARGK2b and an ~99.3% cure of pFA545gen and a 09V transposon mutant library with an ~99.5% cure of pMARGK2b and an ~86.8% cure of pFA545gen. Due to the incomplete loss of the transposase carrying plasmid pFA545gen, subsequent assays were performed in the presence of 5 μg/mL erythromycin to ensure that the genomic insertion of the transposon was maintained. The complexity of both transposon mutant libraries was confirmed using the Bio-TraDIS pipeline, which revealed that the insertions occurred in both strains across the genomes without any obvious hot spotting (see Fig. S1 in the supplemental material). The analysis also identified 265,797 and 175,914 unique transposon insertion sites in the S0385 and the 09V transposon mutant libraries, respectively (Table 1).

**TABLE 1:** Summary of TraDIS data obtained from sequencing transposon mutant input and output libraries

Transposon mutant library	Total reads	Reads with transposon tag (%)	Reads mapped to AM990992 (%)	Unique transposon insertion sites (UIS)	Library saturation (transposon insertion every *n* bp in genes)
S0385-Raw	14,523,982	13,015,971 (89.6)	9,703,026 (74.55)	265,797	10.81
S0385-porcine blood-1	18,256,958	16,237,349 (88.9)	11,281,496 (69.48)	166,075	17.30
S0385-porcine blood-2	14,616,768	12,952,070 (88.6)	8,923,916 (68.90)	180,868	15.88
S0385-porcine blood-3	14,978,172	13,271,260 (88.6)	9,386,389 (70.73)	173,377	16.57
S0385-human blood-1	15,629,643	13,869,490 (88.7)	9,970,236 (71.89)	190,241	15.10
S0385-human blood-2	17,103,125	15,182,720 (88.8)	11,047,499 (72.76)	191,582	14.99
S0385-human blood-3	15,615,069	13,792,965 (88.3)	9,629,625 (69.82)	175,265	16.39
09V-Raw	16,198,809	14,913,221 (92.1)	11,339,428 (76.04)	175,914	15.67
09V-porcine blood-1	18,367,736	16,885,485 (91.9)	13,149,773 (77.88)	120,931	22.79
09V-porcine blood-2	15,740,147	14,256,001 (90.6)	10,492,915 (73.60)	134,732	20.45
09V-porcine blood-3	14,150,768	12,834,082 (90.7)	9,335,365 (72.74)	129,776	21.24
09V-human blood-1	17,749,732	15,915,159 (89.7)	12,283,127 (77.18)	138,172	19.94
09V-human blood-2	17,213,904	15,645,273 (90.9)	11,810,562 (75.49)	139,490	19.76
09V-human blood-3	14,778,446	13,096,888 (88.6)	8,935,862 (68.23)	135,973	20.27

**FIG 1:**
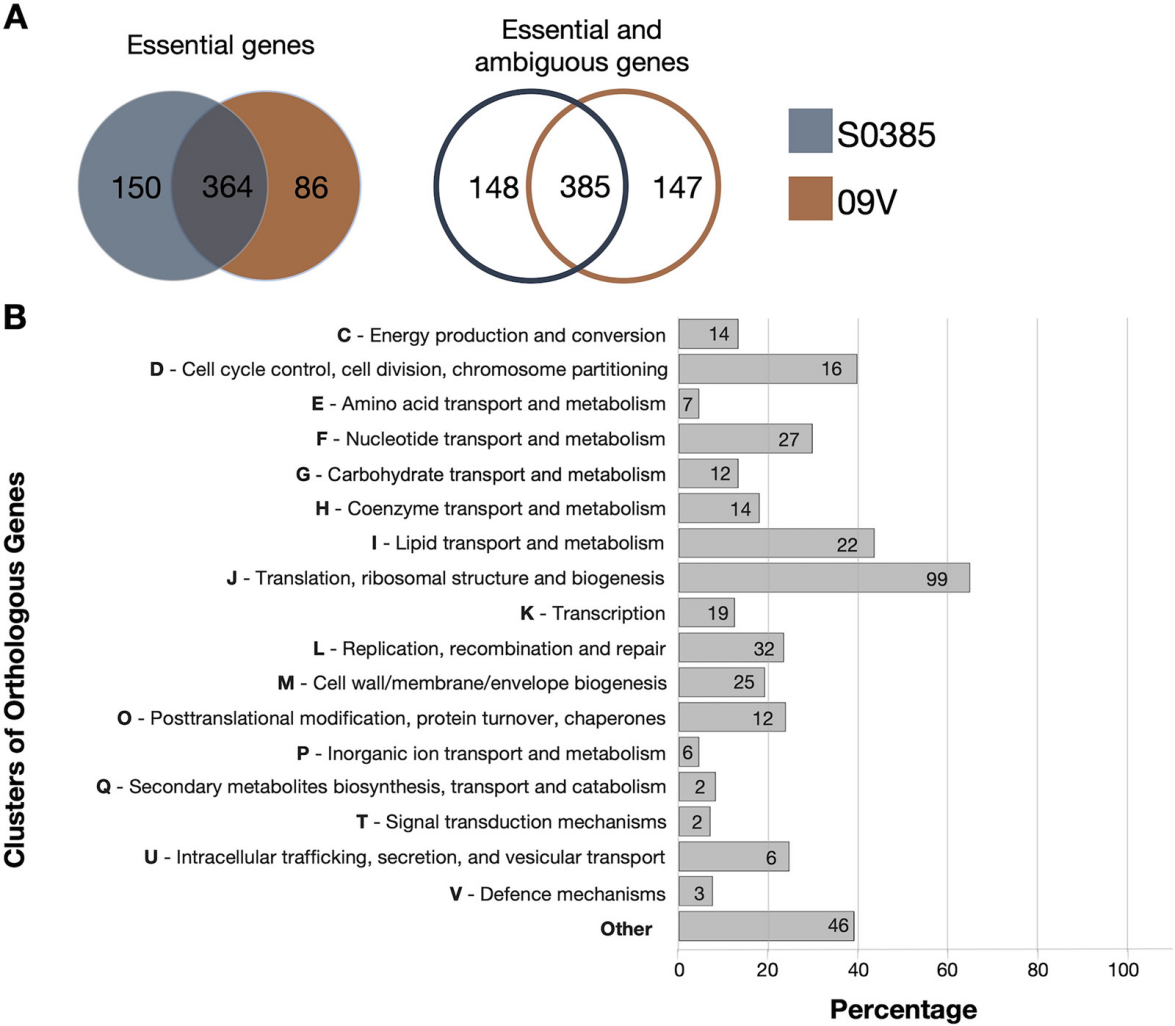
Functional categorization for predicted essential genes (for growth in BHI broth) presented in both ST398 MRSA mutant libraries (S0385 and 09V). (A) Venn diagrams showing the numbers of essential genes and ambiguous genes identified for growth in BHI broth. (B) Functional annotation for the essential genes was conducted with the EggNOG-mapper web service (http://eggnog-mapper.embl.de/) (25). The genes were assigned a functionality based on the COG database, and these groups are illustrated on the *y* axis. The percentages of essential genes identified within each COG in strain S0385 are shown on the *x* axis, with the number (*n*) of genes indicated within each bar. Genes with unknown function and genes within multiple COGs were classified as “other.”

Analysis of the ST398 MRSA raw transposon mutant libraries with the tradis_essentiality TraDIS toolkit script (24) identified essential, ambiguous, and nonessential genes for growth in brain heart infusion (BHI) medium based on the insertion index attributed to each gene. According to the essential and ambiguous changepoints calculated by the script, 514 essential genes and 19 ambiguous genes were predicted for strain S0385, and 450 essential genes and 82 ambiguous genes were predicted for strain 09V (Fig. 1A, Table S3 and S4). Although both transposon mutant libraries had a similar total number of essential and ambiguous genes, they shared a combined list of 364 essential genes (385 genes if both essential genes and ambiguous genes were considered), demonstrating the increased confidence using two closely related strains. Functional annotation of the predicted essential genes presented in both transposon mutant libraries was conducted with the eggNOG-mapper web service (http://eggnog-mapper.embl.de/) (25). The combined essential genes were categorized into 18 clusters of orthologous groups (COGs) (Fig. 1B). Genes involved in translation (COG group J) had the largest number of representatives in the essential gene sets (*n* = 99), followed by genes involved in replication and repair (COG group L) (*n* = 32), nucleotide metabolism and transport (COG group F) (*n* = 27), cell wall/membrane/envelope biogenesis (COG group M) (*n* = 25), and lipid metabolism (COG group I) (*n* = 22). A total of 35 genes were of unknown function, while 9 genes were categorized as belonging to several COGs.

### Survival in porcine blood and human blood.

**FIG 2:**
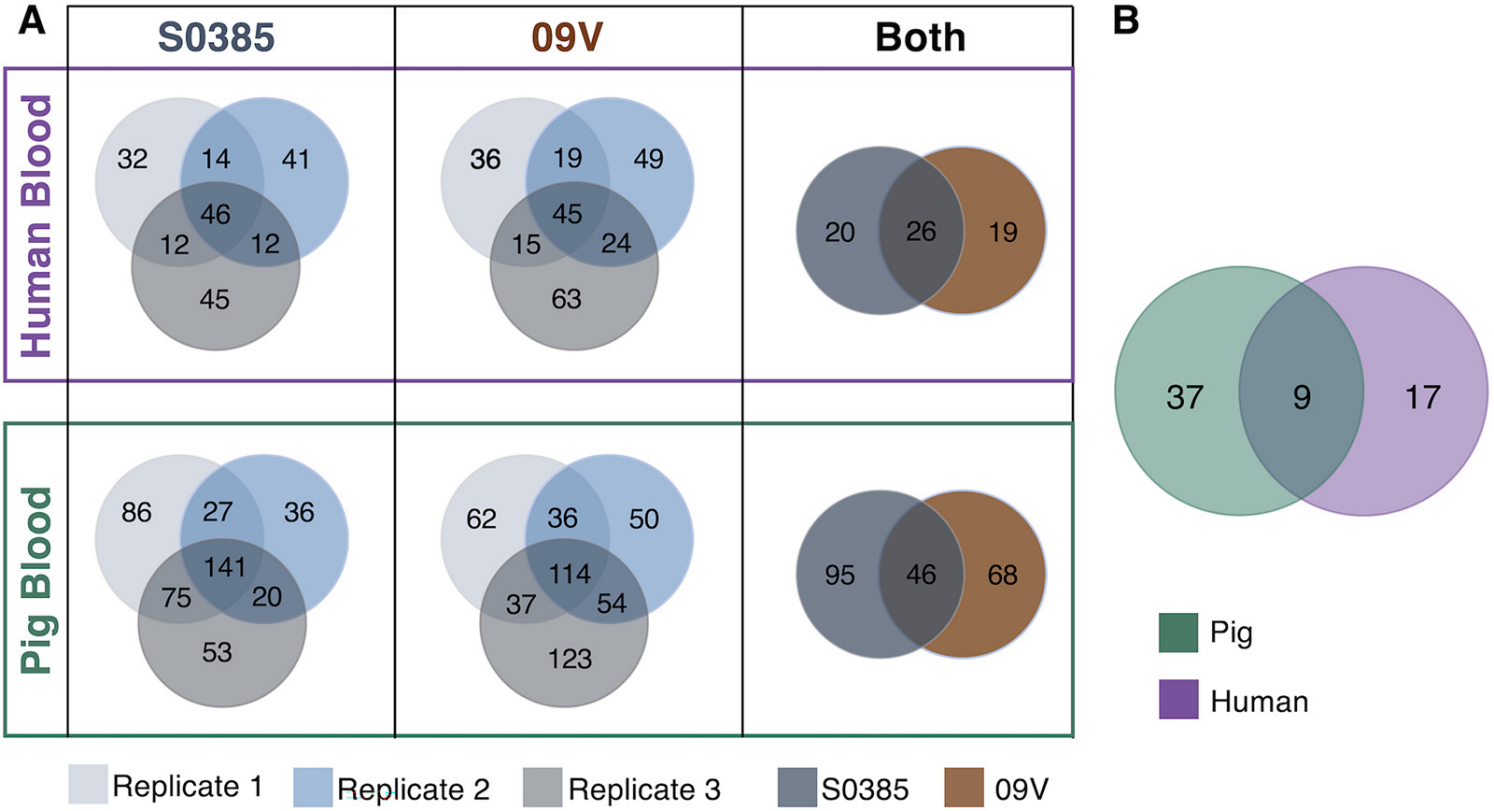
(A and B) Venn diagrams showing (A) the shared number of conditionally essential genes across three biological replicates in S0385, 09V, and both strains for survival in human and porcine blood and (B) the number of shared genes conditionally essential for survival in both human and porcine blood across both strains.

As illustrated in Fig. S2, in the blood survival assay, MRSA ST398 transposon mutant libraries as inputs were passed through 3 different porcine blood and 3 different human blood assays. DNA extracted from input transposon mutant libraries and from output transposon mutants recovered from post-blood assays were prepared and sequenced using the TraDIS approach. Output transposon mutants from each assay condition were compared with the corresponding input transposon mutant library. A log_2_ fold change (log2FC) of ≤ –2 and a *P* value of <0.05 (26) for an individual transposon mutant were considered significantly important (conditionally essential) for blood-bacterial growth. Reproducibility was observed between the 3 biological replicates for both strains in human (Pearson’s correlation, S0385: *R* ≥ 0.64; 09V: *R* ≥ 0.56) and porcine blood (Pearson’s correlation, S0385: *R* ≥ 0.77; 09V: *R* ≥ 0.69), providing confidence in the results of the screen (Fig. S3). When all three essential gene lists for porcine blood survival were compared, 141 shared conditionally essential genes were revealed using the S0385 transposon mutant library, and 114 were revealed using the 09V mutant library. There were 46 conditionally essential genes identified by considering both of the MRSA ST398 transposon mutant libraries. Similarly, 46 conditionally essential genes were identified to be shared by the 3 human blood assays using the S0385 transposon mutant library, and 45 were identified using the 09V transposon mutant library. A combined list of 26 conditionally essential genes was identified for bacterial growth in human blood. Finally, these two combined conditionally essential gene lists were compared, resulting in a list of 9 genes conditionally essential for S. aureus ST398 growth in both porcine and human blood (Table 2 and Fig. 2). The genes conditionally essential for porcine blood survival were distributed in 16 COG groups, with genes involved in amino acid transport and metabolism (COG group E) having the largest number (*N* = 8), closely followed by genes involved in nucleotide metabolism and transport (COG group F, *N* = 6) and inorganic ion transport and metabolism (COG group P, *N* = 5). Similarly, COG group E also had the largest number of representatives (*N* = 6) in the genes conditionally essential for human blood survival, which were distributed in 12 COG groups. In the nine genes conditionally essential for S. aureus growth in both porcine and human blood, three were involved in energy production and conversion (COG group C). Of note, 61.5% (16/26) of genes conditionally essential for porcine blood survival and 60.4% (29/48) of the genes conditionally essential for human blood survival could be assigned to COGs related to the general “metabolism” categories, i.e., groups C, E, F, G, H, I, and P (Table 2).

**TABLE 2:** Genes predicted to be conditionally essential for survival in human and porcine blood for both ST398 MRSA strains in all biological replicates^*a*^

Gene ID	Gene name	Description	COG^*b*^	Host	Gene location
SAPIG1058	*qoxA*	Putative quinol oxidase subunit 2	C	Human/pig	1110272-1111372
SAPIG1464	*aroA*	3-phosphoshikimate 1-carboxyvinyltransferase	E	Human	1572201-1573499
SAPIG1465	*aroB*	3-Dehydroquinate synthase	E	Human	1573509-1574573
SAPIG1043	*menB*	1,4-Dihydroxy-2-naphthoyl-CoA synthase	H	Human	1091740-1092561
SAPIG1054	*fmtA*	Teichoic acid d-Ala esterase FmtA	V	Human/pig	1105865-1107058
SAPIG1445	*gpsB*	Cell division protein GpsB	D	Human	1553361-1553705
SAPIG1603	*aroK*	Shikimate kinase	F	Human	1690753-1691277
SAPIG2002	*purB*	Adenylosuccinate lyase	F	Human/pig	2079231-2080526
SAPIG1466	*aroC*	Chorismate synthase	E	Human	1574599-1575765
SAPIG1113	*ctaB*	Putative protoheme IX farnesyltransferase	O	Human/pig	1168517-1169428
SAPIG1844	*menE*	O-succinylbenzoic acid-CoA ligase	H	Human	1953769-1955247
SAPIG1040	*pchA*	Salicylate biosynthesis isochorismate synthase	HQ	Human	1088086-1089297
SAPIG1617	*pbp2*	Penicillin-binding protein 2	M	Human	1699629-1701704
SAPIG0997		Hypothetical protein	Q	Human	1048526-1049332
SAPIG2568	*fbp*	Fructose-1,6-bisphosphatase	G	Human/pig	2654516-2656480
SAPIG1057	*qoxB*	Cytochrome *aa*3 quinol oxidase, subunit I	C	Human/pig	1108284-1110272
SAPIG1661	*aroE*	Shikimate 5-dehydrogenase	E	Human	1742176-1742982
SAPIG1660	*yhbY*	Ribosome assembly RNA-binding protein YbhY	J	Human/pig	1741882-1742172
SAPIG1790	*aroF*	3-Deoxy-7-phosphoheptulonate synthase	E	Human	1891461-1892552
SAPIG1303	*glpD*	Aerobic glycerol-3-phosphate dehydrogenase	C	Human/pig	1369044-1370717
SAPIG2013	*rutB*	Peroxyureidoacrylate/ureidoacrylate amidohydrolase RutB	Q	Human	2091997-2092557
SAPIG0725	*fepC*	Ferric enterobactin transport ATP-binding protein FepC	HP	Human	760373-761170
SAPIG0017	*purA*	Adenylosuccinate synthetase	NA	Human/pig	22441-23724
SAPIG0883	*aroD*	3-Dehydroquinate dehydratase	E	Human	927295-928011
SAPIG1300	*glpP*	Glycerol uptake operon antiterminator regulatory protein, GlpP	K	Human	1365435-1365968
SAPIG1842	*menC*	NAAAR, *N*-acylamino acid racemase	M	Human	1952763-1953764
SAPIG1200	*carB*	Carbamoyl-phosphate synthase, pyrimidine-specific, large chain	F	Pig	1252798-1255971
SAPIG0844	*whiA*	Putative sporulation transcription regulator WhiA	K	Pig	888976-889920
SAPIG1388	*pstA*	Phosphate ABC superfamily ATP-binding cassette transporter, membrane protein	P	Pig	1461220-1462137
SAPIG1397	*dapB*	4-Hydroxy-tetrahydrodipicolinate reductase	E	Pig	1471812-1472534
SAPIG0349		Hypothetical protein	S	Pig	377858-379024
SAPIG1197	*pyrB*	Aspartate carbamoyltransferase catalytic subunit	F	Pig	1249530-1250411
SAPIG1247		Hypothetical protein	M	Pig	1303171-1303383
SAPIG1056	*qoxC*	Cytochrome *aa*3 quinol oxidase, subunit III	C	Pig	1107689-1108294
SAPIG2312		Hypothetical protein	NA	Pig	2388248-2388565
SAPIG1877		Hypothetical protein	NA	Pig	1977621-1977791
SAPIG0928		Hypothetical protein	P	Pig	967090-968244
SAPIG1587		Putative geranyltranstransferase	H	Pig	1676754-1677635
SAPIG1386	*phoU*	Phosphate uptake regulator	P	Pig	1459674-1460315
SAPIG1332	*thrB*	Homoserine kinase, ThrB	F	Pig	1396368-1397282
SAPIG1136	*rnhC*	Ribonuclease HIII	L	Pig	1190466-1191422
SAPIG1329	*yclM*	Aspartokinase 3	E	Pig	1392446-1393828
SAPIG1671	*accB_2*	Biotin carboxyl carrier protein of acetyl-CoA carboxylase	I	Pig	1752162-1752611
SAPIG1395	*asd*	Aspartate semialdehyde dehydrogenase	E	Pig	1469937-1470926
SAPIG1199	*carA*	Carbamoyl-phosphate synthase small chain	F	Pig	1251705-1252805
SAPIG1623	*cshB*	DEAD-box ATP-dependent RNA helicase CshB	JKL	Pig	1705813-1707159
SAPIG1389	*pstC*	Phosphate ABC superfamily ATP-binding cassette transporter, membrane protein	P	Pig	1462139-1463065
SAPIG2255	*lacA*	Galactose-6-phosphate isomerase subunit LacA	G	Pig	2342307-2342735
SAPIG0699		Hypothetical protein	S	Pig	733268-733492
SAPIG1320		Hypothetical protein	S	Pig	1384832-1385536
SAPIG2159	*prmC*	Modification methylase hemk family protein	J	Pig	2235773-2236606
SAPIG1398	*dapH_3*	2,3,4,5-Tetrahydropyridine-2,6-dicarboxylate *N*-acetyltransferase	E	Pig	1472561-1473280
SAPIG1387	*pstB*	Phosphate ABC superfamily ATP-binding cassette transporter, ABC protein	P	Pig	1460322-1461173
SAPIG0966		DNA segregation ATPase ftsk/spoIIIE	D	Pig	1013337-1014722
SAPIG1366		Prephenate dehydrogenase	E	Pig	1438573-1439403
SAPIG1066	*purQ*	Putative phosphoribosylformylglycinamidine synthase I	F	Pig	1116697-1117368
SAPIG1606		Hypothetical protein	NU	Pig	1692211-1692645
SAPIG1335	*lysP*	Lysine-specific permease	E	Pig	1398967-1400421
SAPIG1469	*menG_2*	Demethylmenaquinone methyltransferase	H	Pig	1578060-1578785
SAPIG1700	*secDF*	Protein translocase subunit SecDF	U	Pig	1784754-1787033
SAPIG1211		Hypothetical protein	NA	Pig	1265866-1266051
SAPIG1525		Virulence-associated E family protein	S	Pig	1626619-1629066
SAPIG1379	*nikE*	Nickel import ATP-binding protein NikE	E	Pig	1452334-1453035
SAPIG2057	*hisC_2*	Histidinol-phosphate aminotransferase	E	Pig	2128614-2129900

a The gene ID and gene location correspond to the genome annotation of strain S0385 (accession AM990992), with gene names and gene functions updated from a new Prokka annotation.

b NA, not assigned.

Additionally, we have included gene *SAPIG1525* in the list of genes conditionally essential for S. aureus growth in porcine blood for the subsequent analysis. Although the *P* value for the change in *SAPIG1525* transposon mutant abundancy was greater than 0.05 (*P* = 0.5) in all 3 biological replicates in the porcine blood survival assay using the S0385 transposon mutant library, a *P* value of <0.05 was observed in 3 biological replicates using the 09V transposon mutant library, and a log_2_FC of ≤ of −2 for the transposon mutant was observed in all 3 biological replicates for both strains S0385 and 09V in the porcine blood survival assay.

### Conservation of genes conditionally essential for survival in blood.

All 26 genes identified to be important for blood survival in human blood are part of the core genome of CC398 (present in at least 99% strains of the 1,180 genomes evaluated). The identified genes were present within the S0385 and 09V core genome with a single-copy number except for the *hflX* gene that was duplicated in both strains. Of the 47 genes (including *SAPIG1525*) identified to be important for survival in porcine blood, 45 are part of the core genome of CC398 (present in at least 99% of the 1,180 genomes evaluated). Genes *SAPIG0966* and *SAPIG1525* were identified as part of the accessory genome. These genes were identified and investigated as potential candidates for host-adaptive function.

**FIG 3:**
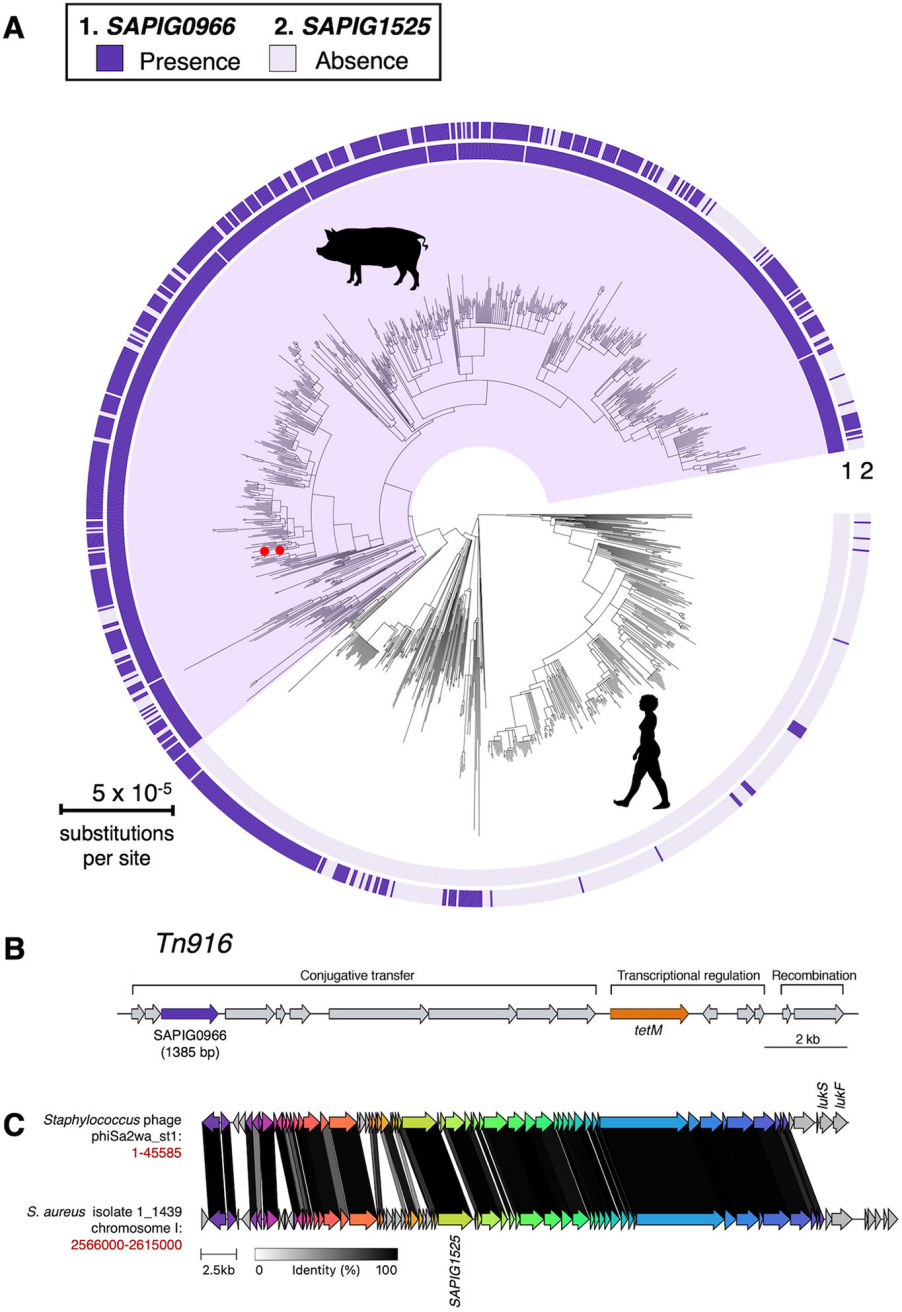
Gene *SAPIG0966* is associated with the livestock clade. (A) A maximum likelihood phylogeny of 1,180 isolates of CC398, rooted using an outgroup as previously described (19). Purple shading indicates the livestock-associated clade. LA-MRSA ST398 strains S0385 and 09V are indicated by red dots. Outer rings describe the presence of genes essential for survival in porcine blood (1) *SAPIG0966* and (2) *SAPIG1525*. (B) Tn*916* transposon in CC398 (based on reference genome 1_1439; NZ_LT992456.1), with annotations based on previous studies (19, 38, 60). Gene *SAPIG0966* (locus tag DXE58_RS01795 in reference genome 1_1439) is located in the conjugative transfer region of the Tn*916* transposon. (C) Comparison of *SAPIG1525* containing phage MGE identified in this study with a previously described phage phiSa2wa_st1 (GenBank MF580410.1) carrying Panton-Valentine leucocidin (PVL).

To investigate gene presence and absence in our genome collection, genes *SAPIG0966* and *SAPIG1525* were searched with BLAST nucleotide against all the 1,180 *de novo* assemblies. All genes with 85% or higher nucleotide identity and 80% or higher length match were annotated as present on a reference-mapped phylogeny (Fig. 3A). Gene *SAPIG0966* is only present in the livestock clade (*N* = 698), which suggests that this gene could have a potential role in livestock adaptation. The *SAPIG1525* gene was identified in 639 strains present in both the human and livestock clades.

Gene *SAPIG0966* was identified to be located on a previously identified Tn*916* transposon carrying a tetracycline resistance gene (Fig. 3B), which has been stably vertically inherited within the CC398 livestock clade for 57 years (19). *SAPIG1525* was identified to be located on a phage containing the same genes and the order of the genes across all strains that have *SAPIG1525* (Fig. 3C). This phage mapped closely to a previously described phage, phiSa2wa_st1, carrying Panton-Valentine leucocidin (PVL) (27).

### Construction of targeted gene deletion mutants.

Two genes (*SAPIG0997* and *aroA*) essential for bacterial survival in human blood, two genes (*SAPIG0966* and *SAPIG1525*) essential for bacterial survival in porcine blood, and four genes (*fmtA*, *purA*, *ctaB*, and *yhbY*) essential for survival in both conditions were selected for the construction of deletion mutants in both ST398 MRSA strains S0385 and 09V. Tn*916*-mediated element (Fig. 3B) carrying the gene *SAPIG0966* was deleted in its entirety in both strains. Multiple attempts failed to delete gene *aroA* and *SAPIG0966* in either strain, for reasons unknown. Other selected genes/elements were successfully deleted in both strain backgrounds. The targeted gene deletion mutants constructed are listed in Table S2.

### Blood survival assay for targeted gene deletion mutants.

**FIG 4:**
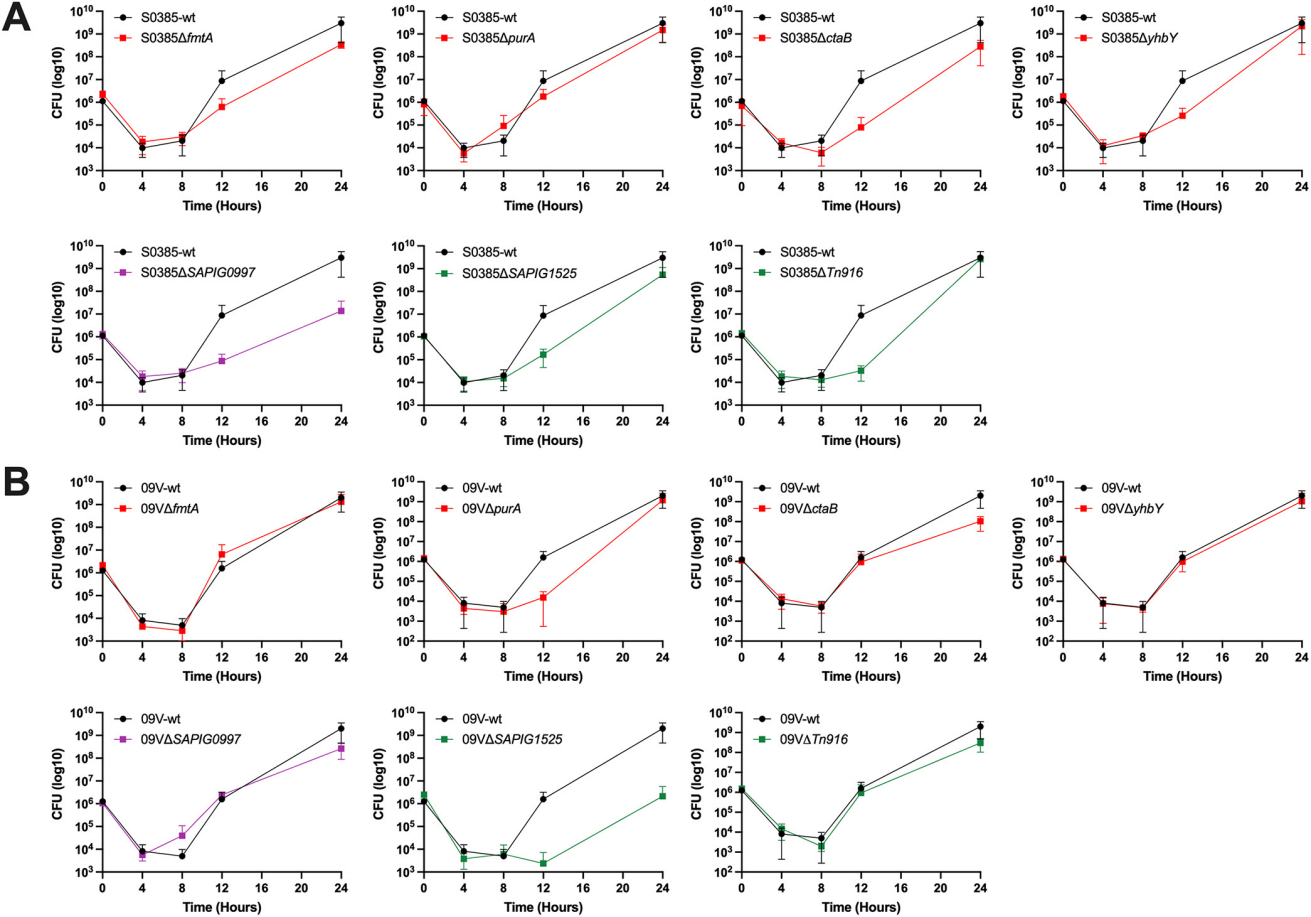
Growth curves in porcine blood for ST398 MRSA strains. (A) Growth curves for S0385 strains and single-gene deletion mutants. (B) Growth curves for 09V strains and single-gene deletion mutants. The wild-type strains are represented with a black growth curve. Strains represented with a red line are mutants with genes predicted to be essential for growth in both porcine and human blood deleted. Strains represented with a purple line are mutants with genes predicted to be essential for growth in human blood deleted. Strains represented with a green line are mutants with genes predicted to be essential for growth in porcine blood deleted.

**FIG 5:**
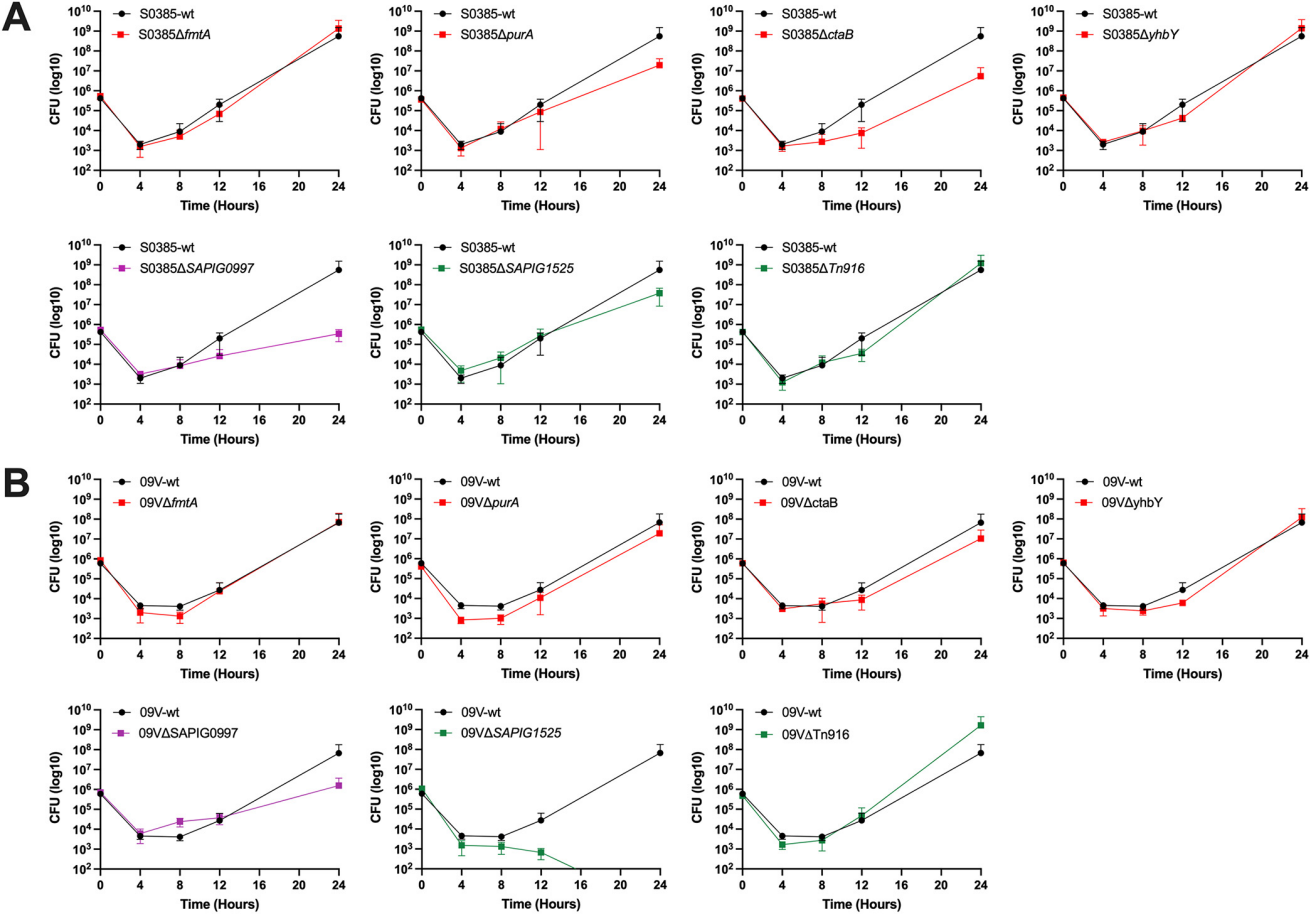
Growth curves in human blood for ST398 MRSA strains. (A) Growth curves for S0385 strains and single-gene deletion mutants. (B) Growth curves for 09V strains and single-gene deletion mutants. The wild-type strains are represented with a black growth curve. Strains represented with a red line are mutants with genes predicted to be essential for growth in both porcine and human blood deleted. Strains represented with a purple line are mutants with genes predicted to be essential for growth in human blood deleted. Strains represented with a green line are mutants with genes predicted to be essential for growth in porcine blood deleted.

To verify the conditional essentiality of the identified genes for ST398 MRSA growth in blood, the ability to survive and recover in blood was tested for the wild-type strains and their gene deletion mutants during incubation at 37°C for 24 h. In both human blood and porcine blood, there was an initial decrease in bacterial cell counts, up to 8 h for all the tested strains (Fig. 4 and 5). The rate of the initial decrease was comparable between the wild-type strains and their gene deletion mutants, while the rate of recovery varied in different strains.

The results showed that all strains managed to recover bacterial cell counts after the initial killing in fresh blood, suggesting that none of the genes chosen for deletion was indispensable for ST398 MRSA to survive in the blood used in this study. Although some targeted gene deletion mutants seemingly recover slower than their wild-type strains (Fig. 4 and 5), multiple unpaired *t* tests with Bonferroni-Dunn correction for *P* values did not find a significant difference (*P* > 0.05) in any of the targeted gene deletion mutants in either blood type tested at any of the time points. However, data showed that the bacterial population started to recover from 4 h (or 8 h) after incubation and grow exponentially to 24 h (the end of the experiment). To compare the rate of the exponential growth between wild-type and targeted gene deletion mutant strains, we calculated their doubling time when cultured in blood between the incubation time of 4 and 24 h (Table S5). The analysis showed that Δ*ctaB* mutants grew slightly slower than the wild-type strains in both blood types, confirming that the gene was beneficial at least for ST398 MRSA growth in porcine and human blood. For Δ*fmtA*, Δ*purA*, and Δ*yhbY* mutants, the analysis gave inconsistent results (Table S5), indicating that these genes were not truly conditionally essential as predicted by TraDIS. For instance, in comparison to the wild-type strains, S0385Δ*fmtA* grew slower in porcine blood, but faster in human blood; 09VΔ*fmtA* grew marginally faster in porcine blood but slower in human blood. Targeted gene deletion mutants Δ*SAPIG1525* and ΔTn*916* grew slower in porcine blood but not in human blood, supporting the TraDIS prediction that they were important for ST398 MRSA growth in porcine blood. Although *SAPIG09097* was identified by TraDIS as essential for ST398 MRSA growth in only the human blood, the targeted gene deletion mutants grew slower in both porcine and human blood compared with the wild-type strains. Overall, the doubling time analysis also showed that the strains tested generally grew slower in human blood than in porcine blood (Table S5), indicating that ST398 MRSA has not been fully adapted to the human blood environment.

## DISCUSSION

LA-MRSA CC398 has gained much attention during the last decade due to being associated increasingly with human infections, which are sometimes severe (8, 28–30). It was suggested that CC398 MRSA originated in humans as methicillin-susceptible S. aureus and became established in livestock after the acquisition of mobile genetic elements (MGEs) associated with both tetracycline and methicillin resistance genes (*tetM* and *mecA*) and the loss of genes associated with human immune evasion (the immune evasion gene cluster) (17, 19). However, previous studies comparing the genomes of CC398 isolates from humans and livestock failed to identify genetic signatures responsible for human adaptation that could lead to diseases, including bacteremia. In this study, using the TraDIS approach, we identified 47 genes and 26 genes that were important (conditionally essential) for ST398 MRSA survival in porcine blood and human blood, respectively, with 9 genes identified to be important for bacterial survival in both blood types (Table 2).

All genes identified as conditionally essential for survival in human blood were found to be part of the core genome of CC398, which suggests that all members of S. aureus CC398 can cause human bloodstream infection. This agrees with a previous finding that all S. aureus lineages are equally capable of causing bloodstream infection (31). However, two genes (*SAPIG0966* and *SAPIG1525*) that were conditionally essential for CC398 MRSA survival in porcine blood were found to be part of the accessory genome. These genes are carried on two different MGEs (the Tn*916* transposon and a phage element) and are associated with antibiotic resistance and host adaptation, respectively (17, 19). MGEs carrying antibiotic resistance genes have been previously reported to have functions other than antibiotic resistance, such as the integrative and conjugative element (ICE) ICE*Sp*23FST81 in Streptococcus pneumoniae, which increases both resistance and virulence (32), and carbapenemase-carrying plasmids (pM5_NDM and pM5_OXA) that have increased the fitness and virulence of the bacterial host (33). To the best of our knowledge, this is the first evidence showing that these MGEs could be involved in the survival of CC398 in porcine blood.

The gene *SAPIG0966* (DNA segregation ATPase FtsK/SpoIIIE) was only present in the CC398 livestock clade, and it was located on a previously described Tn*916* transposon carrying a tetracycline resistance gene (Fig. 3B) (19). The FtsK/SpoIIIE family of DNA translocases is important for synchronizing the late stages of cell division with chromosome segregation, which makes them effectors required for the successful completion of the bacterial cell cycle (34, 35). It was demonstrated that S. aureus requires at least one of the two SpoIIIE/FtsK homologues, which operate in independent pathways to ensure correct chromosome management during cell division (36). Unfortunately, in this study, we were not successful in making the *SAPIG0966* deletion mutants in either strain S0385 or 09V after multiple attempts, and therefore, we were unable to confirm its essentiality in porcine blood survival. Instead, we constructed Tn*916* deletion mutants in both the S0398 and 09V strains. Our phenotypic data showed that both mutants had a significantly slower recovery rate after the initial killing in porcine blood (Fig. 4, Table S5), confirming its importance in porcine blood survival. The stable integration of the Tn*916* transposon could be of benefit to a livestock-associated lineage, since tetracyclines are the most commonly used antimicrobial class in livestock farming (7, 37). In addition, previous studies have suggested that Tn*916* is associated with a low selective burden in the absence of treatment, which could increase its stability (38). Here, we show that Tn*916* could have a secondary function, essential for the survival of livestock-associated isolates in porcine (animal) blood. Although we were not able to investigate the contribution of *SAPIG0966* on its own, the fact that it was the only gene on this MGE identified as essential by TraDIS suggests its pivotal role in porcine blood survival. It may explain the ubiquity of Tn*916* and its stable inheritance in livestock-associated isolates even without the antimicrobial selection pressure.

Gene *SAPIG1525* was annotated as encoding a virulence-associated E family protein, and it was present in both human and livestock clades (Fig. 3A). Virulence-associated protein E (VapE, SeseC_01325) was first identified in Dichelobacter nodosus (39) as part of the *vap* region of D. nodosus that is associated with virulence (40). In the staphylococcal species, the *vapE* gene has been found on a genomic island carrying fusidic acid resistance gene *fusB* in some Staphylococcus lugdunensis (41) and Staphylococcus epidermidis (42) strains. In S. aureus, a *vapE*-like gene has also been identified in the pathogenicity island, SaPI1, which contains the gene for toxic shock syndrome toxin-1 (43). This *vapE*-like gene-encoded protein (GenBank AAL67615.1) shows 35% identity to the aligned sequence in the VapE protein in D. nodosus (GenBank AAB00938.1) and 36% identity to the aligned sequence in the *SAPIG1525*-encoded protein (CAQ49949.1) identified in this study. VapE was suggested to be important in the pathogenesis of Streptococcus equi ssp. *zooepidemicus* ATCC35246 (Sz35246) causing serious diseases in pigs (44). In CC398 MRSA, our analysis of the population structure regarding *vapE* strongly suggests its role in livestock (mainly pig) adaptation. *SAPIG1525* deletion mutants in both S0398 and 09V strains showed a significantly slower recovery after the initial killing in porcine blood (Fig. 4), confirming its importance in porcine blood survival. Further analysis revealed that the gene *SAPIG1525* was located on a phage element which showed partial similarity to the previously identified phage phiSa2wa_st1 (GenBank MF580410.1) encoding PVL (27) (Fig. 3C).

Apart from the two genetic elements discussed above, other conditionally essential genes for blood survival, such as *fmtA*, *purA*, *purB*, and *ctaB*, could also be of interest. These genes have been previously studied (45–48) and were identified in this study as conditionally essential for ST398 MRSA survival in both porcine blood and human blood. The methicillin resistance factor, *fmtA*, was found to be a core member of the cell wall stimulon and was shown to interact with teichoic acids (46). Most recently, FmtA was found to hydrolyze the ester bond between d-Ala and the backbone of teichoic acids in the S. aureus cell envelope, suggesting that it functions as a modulator of teichoic acid charge and may be involved in S. aureus cell division, biofilm formation, autolysis, and colonization (47). FmtA was also identified as essential for ST398 MRSA growth in porcine blood (22). The *purA* and *purB* genes encode proteins that are part of the *de novo* biosynthetic pathways for purines. They were found to be required for growth on Mueller-Hinton (MH) agar containing human blood and to contribute significantly to S. aureus pathogenesis in a zebrafish systemic infection model (48). The gene *ctaB* encodes the membrane protein CtaB, which is a protoheme IX farnesyltransferase involved in the synthesis of the heme containing terminal oxidases of the bacterial respiratory chain (49). It was reported that deletion of *ctaB* caused decreased transcription of several virulence genes and significantly downregulated the transcription of 20 ribosomal genes and 24 genes involved in amino acid biosynthesis (45). Deletion of *ctaB* also was shown to attenuate growth and virulence in mice but enhance pigment production and formation of quinolone-tolerant persister cells (45).

To confirm the conditional essentiality of the genes identified by TraDIS mutagenesis, we carried out blood survival assays on gene deletion mutants and their wild-type strains. Our data showed that Δ*fmtA* mutants did not seem to have a disadvantage in survival and growth in porcine blood or human blood, suggesting that *fmtA* may not be truly essential for ST398 MRSA survival and growth in the blood tested (Fig. 4 and 5). Similarly, the ability of the Δ*purA* mutants to recover in blood after the initial killing was not significantly affected, although 09VΔ*purA* only started to recover after 12 h in porcine blood (Fig. 4). This indicates that the *purA* gene (or protein product) may not be essential for growth recovery in fresh blood. For Δ*ctaB* mutants, we found that their growth was adversely affected by both porcine and human blood, as indicated by a longer doubling time (Table S5), confirming that gene *ctaB* was indeed conditionally essential for blood survival. Gene *ybhY* (*SAPIG1660*), annotated to encode ribosome assembly RNA-binding protein YbhY, was also deleted in both ST398 MRSA strains. Although the gene was identified as conditionally essential for bacterial growth in both human and porcine blood, the growth pattern of the Δ*ybhY* strains in either blood type was not different from that of the wild-type strains. This indicates that *ybhY* was not essential for growth in the fresh blood tested. Gene *SAPIG0997*, which encodes a hypothetical protein, was found to be conditionally essential for ST398 MRSA survival in human blood. However, our data show that deletion of the gene had an adverse effect on the bacterial survival and growth in both human and porcine blood (Fig. 4 and 5, Table S5).

We must note that our data from the blood survival assay using single-gene deletion mutants did not agree fully with the results from the TraDIS approach, with some genes verified to be conditionally essential and some not. This discrepancy could have been caused by the competition-based nature of the blood survival assay using an entire mutant library in which some mutants might have been outcompeted in growth and therefore misidentified by TraDIS as having the essential gene disrupted. Additionally, without whole-genome sequencing data, we could not rule out possible genomic changes happened during the blood assay that compensated for the function of the deleted genes in these mutants. Our data also showed there were possible individual differences between blood samples from different animals or human donors, as indicated by the different growth patterns for some gene deletion mutants. Therefore, further research is needed to address this inconsistency by identifying possible reasons and improved protocols. One limitation is that these single-gene deletion mutants should be complemented with a plasmid-borne copy of the deleted gene and subjected to the blood survival assay to provide more convincing evidence for the function of the gene. Another limitation of this study is that we did not include CC398 strains from the human clade, which may provide additional evidence for a conclusion on host adaptation and should be investigated in future work.

In conclusion, we successfully generated a genome-wide transposon mutant library in two closely related ST398 MRSA strains and identified a list of genes that were potentially essential for ST398 MRSA survival and growth in human and porcine blood. Many of these genes were related to general metabolism, such as the metabolism of amino acids, nucleotides, and inorganic ions. Importantly, we found that MGEs that carry resistance and virulence genes could have a secondary function in bacterial survival in blood and may be important for host adaptation. In addition to increased resistance, the previously described Tn*916* could also contribute to the survival of livestock-associated isolates in porcine (animal) blood. A virulence-associated phage element was also found to be important for the survival of livestock-associated isolates in porcine blood. These findings seemingly are contradictory to the widely accepted theory that antibiotic resistance in many bacterial species often incurs a fitness cost in the absence of the antibiotic, such as reduced growth rate, competitive ability, or virulence (50, 51). However, MGEs with beneficial functions other than increasing resistance to antibiotics have also been reported in S. pneumoniae and Escherichia coli (32, 33). We believe these findings offer intriguing insight into the role an MGE plays in a pathogen, including host adaptation, which requires further comprehensive investigations to elucidate.

## MATERIALS AND METHODS

### Media and culture conditions.

The bacterial strains and plasmids used in this study are described in Table S1. For routine culture, S. aureus was grown on tryptone soy agar (TSA) (Oxoid, UK) of horse blood agar (Oxoid) or in tryptone soy broth (TSB) (Oxoid). Escherichia coli was grown in lysogeny broth (Oxoid) or on L agar (Oxoid) at 37°C. For selective culture, the antibiotics chloramphenicol, erythromycin, and gentamicin were supplemented in medium as appropriate. For the construction of mutant libraries, brain heart infusion (BHI) broth (Oxoid) was used to culture the S. aureus strains.

### Construction of transposon mutant libraries.

Temperature-sensitive plasmids (22) pMARGK2b (carrying the *mariner* transposon) and pFA545gen (carrying the transposase) were electroporated sequentially into two closely related ST398 MRSA strains, S0385 (BioSample no. SAMEA2272644) (52) and 09.4620.V (BioSample no. SAMEA1708941; referred to as 09V henceforth) (53), resulting in strains S0385pp′ and 09Vpp′. Each strain was cultured overnight at 30°C in BHI broth supplemented with 5 μg/mL erythromycin and 16 μg/mL gentamicin and stored at −80°C in 0.75-mL aliquots mixed with 0.75 mL 50% glycerol.

Transposon mutagenesis was carried out using the newly transformed strains (S0385pp′ and 09Vpp′), as described previously with minor modifications (22). Briefly, for each strain, a 1-mL glycerol aliquot (>10^7^ cells) was inoculated into 100 mL BHI containing 5 μg/mL erythromycin, 5 μg/mL chloramphenicol, and 16 μg/mL gentamicin and incubated at 30°C with shaking until the cultures reached an optical density at 595 nm (OD_595_) of 0.4. Then, cells from 30 mL culture were collected by centrifugation at 3,000 × *g* for 10 min and inoculated in 600 mL BHI containing 5 μg/mL erythromycin prewarmed to 43°C and then incubated at 43°C with shaking. When the cultures reached an OD_595_ of 0.4 again, cells from 30 mL culture were collected by centrifugation at 3,000 × *g* for 10 min and inoculated in 600 mL BHI containing 5 μg/mL erythromycin prewarmed to 43°C, and the cultures were incubated at 43°C for 20 h. The following day, cells from the 30-mL culture were collected by centrifugation and inoculated in 600 mL prewarmed BHI containing 5 μg/mL erythromycin, and the cultures were incubated at 43°C for 20 h. The same procedure was repeated once more, resulting in a 3^rd^-generation transposon mutant library. To determine the size of the transposon libraries (i.e., the number of transposon mutants) and the loss of the plasmids, each day the cultures were serially diluted and plated on TSA supplemented with 5 μg/mL erythromycin, TSA supplemented with 10 μg/mL chloramphenicol, and TSA supplemented with 16 μg/mL gentamicin and incubated at 37°C overnight. Transposon mutants were stored in 1.5-mL (~10^9^ cells) 25% glycerol aliquots at −80°C until further use. For each transposon mutant library, DNA from ~10^9^ transposon mutants was extracted using a MasterPure Gram-positive DNA purification kit (Epicentre) and stored as an input pool at −20°C.

### Whole-blood survival assay for transposon mutant libraries.

Heparinized human whole blood from three healthy human donors and heparinized porcine whole blood from three healthy euthanized pigs were used in the whole-blood survival assay for the transposon mutant libraries. Human blood donors were informed of the purpose of the study, and written consent was obtained. Porcine whole blood was collected from a local abattoir with the pig owner’s permission. Ethical review was undertaken at the Department of Veterinary Medicine, University of Cambridge (CR76).

Whole-blood survival was carried out as described previously (22). Briefly, 10 mL of each freshly collected blood sample was inoculated with 1 mL transposon mutant library glycerol stock (~10^9^ cells) and incubated for 24 h at 37°C with aeration. Viable cell counts were tested the following day, and 0.5 mL from each blood culture was inoculated into 10 mL BHI supplemented with 5 μg/mL erythromycin, to increase the bacteria/blood cell ratio prior to DNA extraction, and incubated overnight (20 h) at 37°C with aeration. Viable cell counts of each culture again were determined, and DNA from ~10^9^ transposon mutants was extracted and stored as output pools at −20°C.

### Library preparation and sequencing by TraDIS.

For the TraDIS approach, the sequencing libraries were prepared as previously described with modifications (22, 23). DNA (2 μg) from the input and output transposon mutant pools was fragmented to an average size of ~200 bp using a Covaris E210 instrument. The size profile was evaluated with an Agilent 2100 Bioanalyzer on a high-sensitivity DNA1000 chip. Fragmented DNA was prepared for sequencing using a NEBNext Ultra II DNA library prep kit for Illumina (New England Biolabs) according to the manufacturer’s instructions for end repair and adaptor ligation. As recommended by Langridge et al. (23), 100 ng of library DNA was PCR amplified for 22 cycles following THE NEBNext Ultra II DNA library prep kit for Illumina protocol. Amplification utilized the transposon-specific primer (23) (Table S2) and indexing PCR primers from NEBNext multiplex oligos for Illumina (index primer sets 1 and 2). The PCR products were cleaned using 0.9× SPRIselect beads (Beckman Coulter) to remove DNA fragments smaller than 200 bp. The quality of the amplified products was assessed using an Agilent 2100 Bioanalyzer on a high-sensitivity DNA chip and quantified by quantitative PCR (qPCR) with primers P5 and P7. The libraries were pooled in a 1:1 molar ratio and sequenced with a spike-in of 30% PhiX on an Illumina HiSeq 4000 platform on SE50 mode with a custom read 1 primer (GACACTATAGAAGAGACCGGGGACTTATCAGC) (22).

### Analysis of sequencing data.

To identify genes important for survival in blood, raw demultiplexed fastq files were analyzed using the Bio-TraDIS scripts made available by the Sanger Wellcome Trust Institute (https://github.com/sanger-pathogens/Bio-Tradis) (24). Individual transposon mutants with a log_2_ fold change (log_2_FC) of ≤–2 and a *P* value of <0.05 in frequency (26) were considered significantly important (essential) for blood-bacterial growth. Mutants with a log_2_FC of ≥2 and a *P* value of <0.05 were considered overrepresented under the assay condition but were not further investigated in this study. Reproducibility was assessed using Pearson’s correlation on all overlapping genes between the 3 biological replicates on the log_2_FC. Essential genes identified by all 3 porcine blood assays were extracted to obtain a list of genes essential for bacterial growth in porcine blood. Similarly, essential genes identified by all 3 human blood assays were extracted to obtain a list of genes essential for bacterial growth in human blood. Finally, essential genes present in both lists were extracted to obtain a list of genes essential for S. aureus growth in both porcine and human blood.

### Genomic characterization of genes essential for survival in blood.

All genes identified to be essential in blood survival were investigated using a previously published collection of 1,180 CC398 genomes from 14 host species, isolated in 28 different countries, with a wide temporal scale (1993 to 2018) (19). We used sequence data from all isolates to generate *de novo* assemblies and accessed the genome quality as previously described in reference 19. All complete *de novo* assemblies were annotated using Prokka v1.13.3 (54). Pan-genome analysis was conducted using Prokka-annotated assemblies in Roary (55), with minimum protein BLAST identity at 95% and the minimum percentage for a gene to be considered core at 99% without paralog splitting. All genes with known function were identified in the gene presence-absence table using the gene name and gene function description. The genes which were initially not identified, including all hypothetical proteins, were characterized using BLASTp against the pan-genome file generated with Roary to identify corresponding gene names.

The reference-mapped assemblies were generated using Bowtie2 v1.2.2 against the reference genome S0385 (GenBank accession no. AM990992) (52, 56). Recombination was detected in the reference-mapped alignment using Gubbins v2.3.1 (57); additionally, we masked a region of ~123 kb that was identified as horizontally acquired from an ST9 donor in a previous study (17). We carried out phylogenetic reconstruction for the reference-mapped alignment with RAxML v8.2.4 using the GTR +┌ model and 1,000 bootstraps (58). Sites where >0.1% of genomes showed evidence of recombination or had missing data were excluded from the analysis. The group annotation and rooting were carried out as previously described (19).

Genes identified to be important in porcine blood were associated with MGEs as previously described (19) by (i) physical locations within the *de novo* assemblies and published reference genomes, (ii) correlation in their presence/absence across the CC398 phylogeny, and (iii) investigation of the identified MGEs in the literature and in NCBI (19).

### Construction of S. aureus targeted gene deletion mutants.

Selected gene deletion mutants in LA-MRSA ST398 strains were generated by allelic exchange with the temperature-sensitive vector pIMAY, as previously described (59). The primers used for gene deletion are listed in Table S2. Upstream (AB) and downstream (CD) sequences of the S. aureus gene to be deleted were amplified with primers A/B or C/D using KOD Hot Start DNA polymerase (Merck). The PCR products AB and CD were used as templates to obtain deletion construct AD with primers A/D in a splicing overlap extension PCR. Product AD was digested with restriction enzymes KpnI and SacI and ligated to pIMAY digested with the same enzymes. The resulting plasmids were transformed into E. coli DC10B (a *dcm* deletion mutant of DH10B), allowing the plasmid to be directly transferred into S. aureus strains (59). Plasmid DNA extracted from DC10B was then electroporated into recipient strains to create gene deletion mutants.

### Survival assay for targeted gene deletion mutants in blood.

Heparinized human blood (2 mL) from 3 healthy human donors and heparinized porcine blood (5 mL) from 4 healthy euthanized pigs were used in the blood survival assay for each wild-type strain and its deletion mutants. For each strain, the overnight culture in TSB was adjusted to a turbidity of 0.5 MacFarland standard and inoculated 1:100 in each of the bloods, resulting in an initial inoculum of about 10^6^ CFU/mL. The inoculated blood was immediately mixed and incubated at 37°C with continuous shaking for 24 h. The number of bacterial CFU was determined at different time points (0, 4, 8, 12, and 24 h) after incubation by plating serial 10-fold dilutions with 3 or 4 replicates.

Multiple unpaired *t* tests with Bonferroni-Dunn correction for multiple comparison were performed using GraphPad Prism v9 software to evaluate the difference between the growth of the deletion mutants and their respective wild-type strains in blood at 4, 8, 12, and 24 h. To analyze the recovery growth rate, growth curves for each strain between hours 4 and 24 were also analyzed with GraphPad Prism v9 software. The doubling time was calculated with a nonlinear regression model using an exponential growth equation with a least-squares fit, with outliers eliminated and Y0 constrained to the mean value on the *y* axis at time hour 4.

### Data availability.

TraDIS sequencing data in this study is available in NCBI BioProject no. PRJNA883753 (SRA no. SRR21691651 to SRR21691664).
