# Supplementary material for: High-Throughput Mutagenesis Reveals a Role for Antimicrobial Resistance- and Virulence-Associated Mobile Genetic Elements in Staphylococcus aureus Host Adaptation
Source: Microbiol Spectr. 2023 Feb 23;11(2):e04213-22. doi: 10.1128/spectrum.04213-22 (PMC10101091; doi:10.1128/spectrum.04213-22)
Supplement: Supplemental file 2 — Figures S1-S3. Download spectrum.04213-22-s0002.pdf, PDF file, 0.8 MB [file spectrum.04213-22-s0002.pdf]

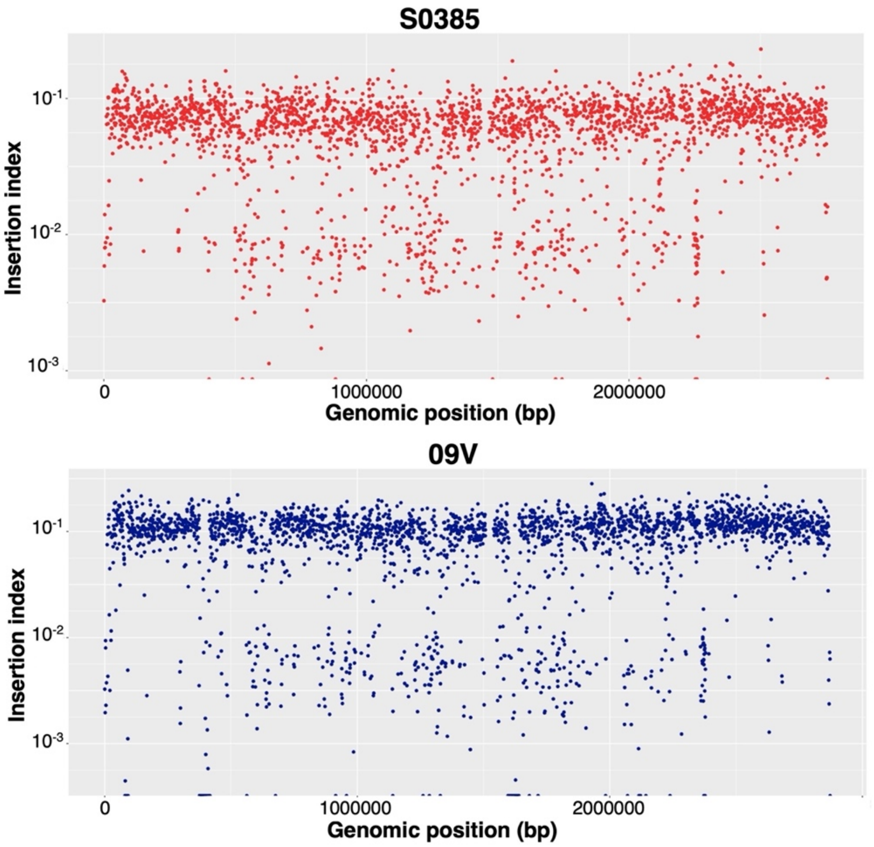

Figure S1: Frequency and distribution of insertions across the genome in the raw mutant libraries. The insertions happened across both genomes: S0385 and 09V without any obvious 'hotspot', indicating the libraries are highly saturated. Y-axis is Insertion Index (log10) which is defined as the number of insertions per gene divided by its length.

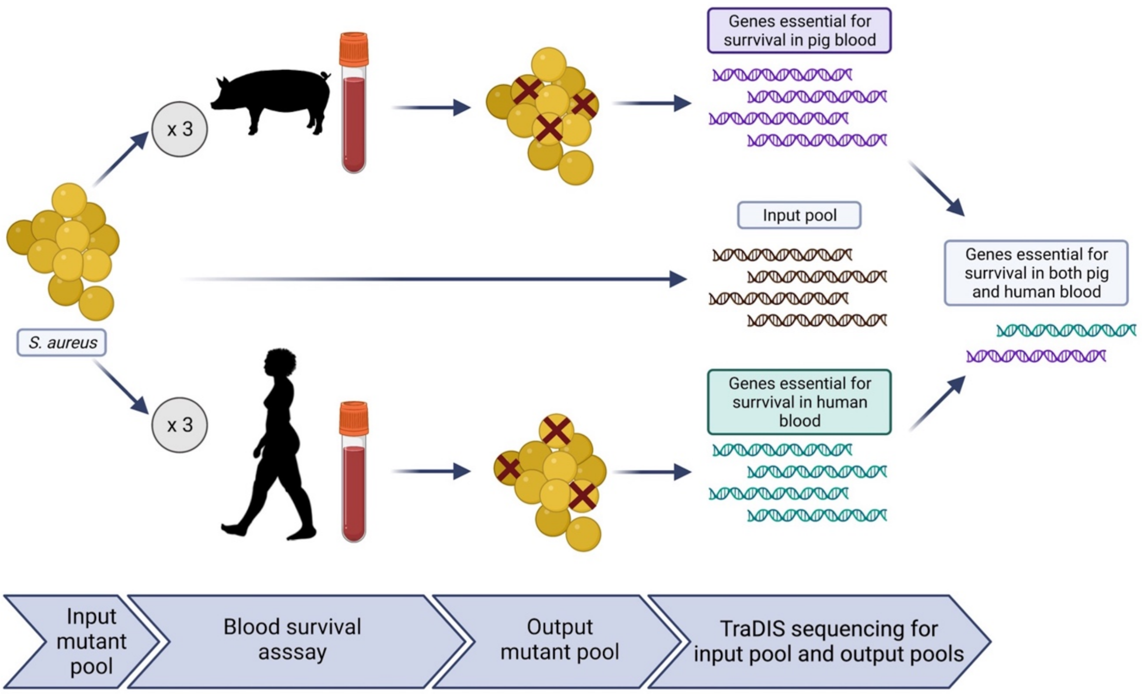

Figure S2: Schematic workflow of blood survival assay and TraDIS analysis. *S. aureus* ST398 transposon mutant library as input was passed through 3 different porcine blood samples and 3 different human blood samples. Output mutant library from each assay condition was compared with the input mutant library and genes essential to each assay condition were identified using the Bio-TraDIS toolkit pipeline.

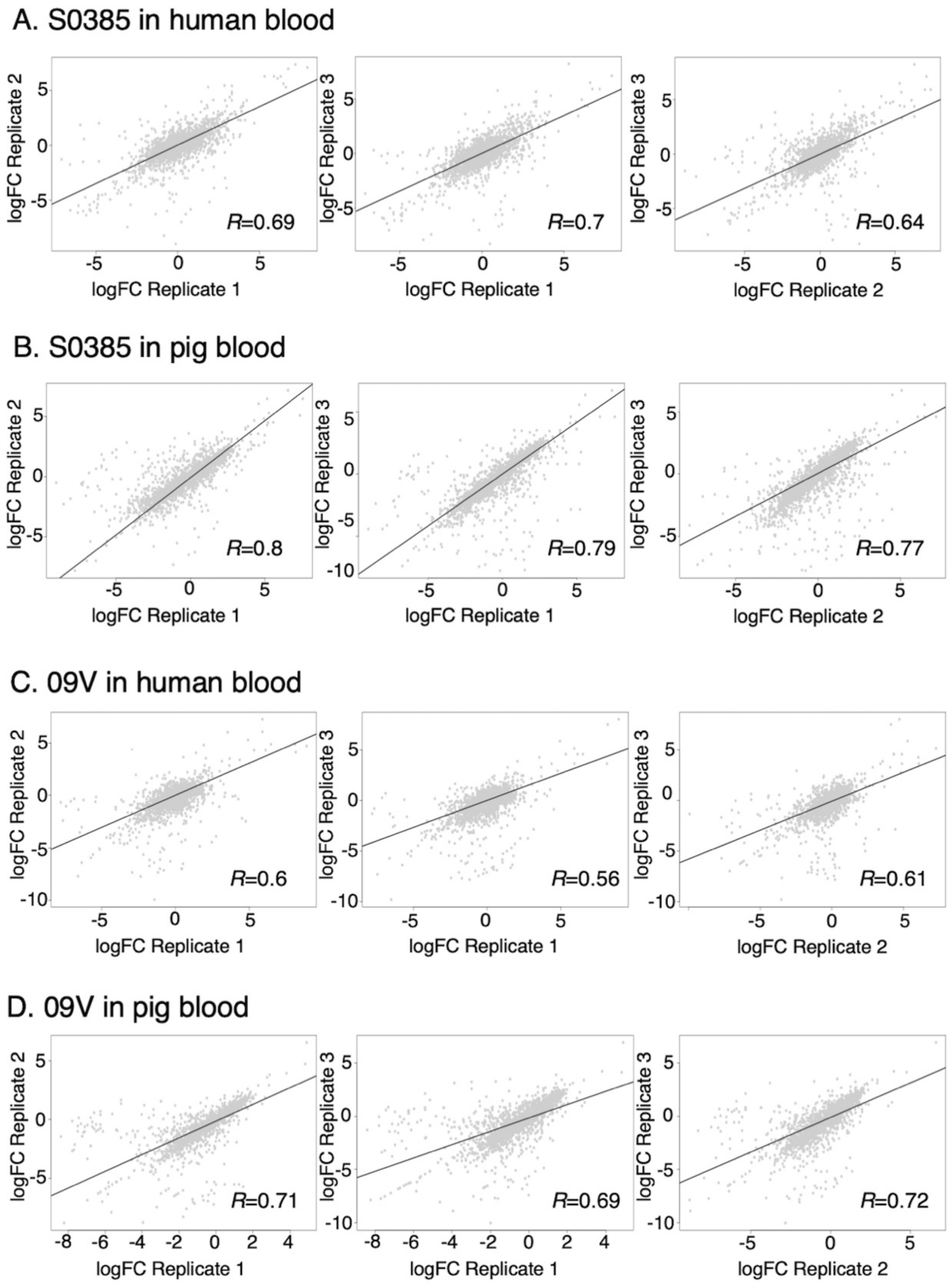

Figure S3: Reproducibility of the TraDIS analysis was assessed using Pearson's correlation on all overlapping genes between the three biological replicates on the log2 fold change (log2FC). Reproducibility was observed between the three biological replicates for SO385 in human (A), and porcine (B) blood, and 09V in in human (C), and porcine (C) blood.
